# Evaluation of Resting State Networks in Patients with Gliomas: Connectivity Changes in the Unaffected Side and Its Relation to Cognitive Function

**DOI:** 10.1371/journal.pone.0118072

**Published:** 2015-02-06

**Authors:** Satoshi Maesawa, Epifanio Bagarinao, Masazumi Fujii, Miyako Futamura, Kazuya Motomura, Hirohisa Watanabe, Daisuke Mori, Gen Sobue, Toshihiko Wakabayashi

**Affiliations:** 1 Brain and Mind Research Center, Nagoya University, Nagoya City, Aichi, Japan; 2 Department of Neurosurgery, Nagoya University School of Medicine, Nagoya City, Aichi, Japan; 3 Department of Rehabilitation, Nagoya Hospital Organization, Nagoya Medical Center, Nagoya City, Aichi, Japan; 4 Department Neurology, Nagoya University School of Medicine, Nagoya City, Aichi, Japan; Hangzhou Normal University, CHINA

## Abstract

In this study, we investigated changes in resting state networks (RSNs) in patients with gliomas located in the left hemisphere and its relation to cognitive function. We hypothesized that long distance connection, especially between hemispheres, would be affected by the presence of the tumor. We further hypothesized that these changes would correlate with, or reflect cognitive changes observed in patients with gliomas. Resting state functional MRI datasets from 12 patients and 12 healthy controls were used in the analysis. The tumor’s effect on three well-known RSNs including the default mode network (DMN), executive control network (ECN), and salience network (SN) identified using independent component analysis were investigated using dual regression analysis. Scores of neuropsychometric testing (WAIS-III and WMS-R) were also compared. Compared to the healthy control group, the patient group showed significant decrease in functional connectivity in the right angular gyrus/inferior parietal lobe of the ventral DMN and in the dorsolateral prefrontal cortex of the left ECN, whereas a significant increase in connectivity in the right ECN was observed in the right parietal lobe. Changes in connectivity in the right ECN correlated with spatial memory, while that on the left ECN correlated with attention. Connectivity changes in the ventral DMN correlated with attention, working memory, full IQ, and verbal IQ measures. Although the tumors were localized in the left side of the brain, changes in connectivity were observed in the contralateral side. Moreover, these changes correlated with some aspects of cognitive function indicating that patients with gliomas may undergo cognitive changes even in the absence of or before the onset of major symptoms. Evaluation of resting state networks could be helpful in advancing our hodological understanding of brain function in glioma cases.

## Introduction

According to a national survey of brain tumor patients in 1989, the most common symptoms include headache (56%), epilepsy (28%), and/or progressive neurological functional loss (68%) [1]. Approximately 30% of patients lack apparent neurological deficits such as speech disturbance or motor weakness. Wide spread use of non-invasive imaging technologies lead to improved diagnosis of patients without or with only minor symptoms, which has further increased this percentage. For these patients, detailed neuropsychometric tests often reveal some deficits in cognitive function including comprehension, performance, executive control, working memory, and attention. Such changes in cognition are often difficult to explain when considering only the damage caused by the tumor in focal regions of brain. To fully understand these changes requires not only topological but also network-based (hodological [2]) approaches.

The importance of cortico-subcortical connections in understanding the patients’ neurological condition has now been widely acknowledged. A widely used method to investigate intrinsic patterns of connectivity within the brain is resting state functional MRI. This method utilizes the fact that spontaneous brain activity in one region within the network is functionally correlated to the activity in another regions, thereby providing a way to identify the spatiotemporal distribution of the so-called resting state network (RSN) [3–5]. Resting state fMRI requires no explicit task for the participant and is therefore highly suitable for examining cognitive changes in the patient population (e.g. in presurgical planning [6,7]), who might not be as responsive as healthy controls in some task designs. Kokkonen et al [8] reported that resting state fMRI showed almost equal power to task-fMRI for the detection of the sensorimotor area in patients with brain tumor. Task-fMRI can be challenging for patients with paresis or attention deficit. Therefore, using resting state fMRI could reduce risk of type 1 error for pre-surgical identification of functionally dominant area.

In the resting state fMRI study, three fundamental networks have been proposed as the underlying neural basis of consciousness, including the default mode network (DMN), the executive control network (ECN), and the salience network (SN) [9,10]. The DMN—which includes the posterior cingulate cortex, the ventromedial prefrontal cortex (VMPFC), the angular gyrus, and the medial temporal lobe, is the most studied network, can be readily detected in resting state fMRI, and is highly reproducible across participants. Its precise function is still largely unknown, but the individual regions comprising it are hypothesized to be involved in stimulus-independent thoughts [11], introspection [12], integration of autobiographical, self-monitoring, and related social cognitive functions [13]. Alterations of the DMN have been reported in several neurological conditions including Alzheimer’s disease, autism, and schizophrenia [14–17]. While the DMN is mostly deactivated during task performance, cognitively demanding tasks activate the ECN. ECN’s key nodes include the dorsolateral prefrontal cortex (DLPFC) and posterior parietal cortex. It is critical for active maintenance and manipulation of information in working memory, and for judgment and decision-making in the context of goal-directed behavior [18].

The last of the 3 networks is the salience network consisting of the anterior insula and the anterior cingulate cortex. The salience network plays a role in switching between activation and deactivation of the other two large scale networks [19].

Network studies of brain tumor cases are very rare and only a few number of papers have been published on this topic so far [20–22]. Harris et al examined the DMN with pseudo-resting state fMRI (i.e., using the datasets derived from task-based fMRI) and showed that the DMN integrity decreased in glioma cases [20]. The significant predictors of the changes in DMN integrity were WHO grade and the location of lesions. On the other hand, the effect of lesion size was insignificant. Esposito et al studied the DMN with glioma cases using deactivation profiles of the language task datasets. They found increased DMN connectivity in the hippocampal lesions but decreased connectivity in the VMPFC. In addition, they observed decrease in connectivity in low grade gliomas instead of high grade gliomas. These two reports were in agreement in terms of the DMN changes in glioma patients, though some differences existed in the details. Recently, Xu et al showed that decrease in network’s global efficiency correlated with intellectual decline in glioma patients.

In this study, we used resting state fMRI and investigated the above-mentioned RSN in glioma patients with little or no neurological functional deficits. We hypothesized that long distance connection, especially between hemispheres, would be affected by the presence of the tumor which would manifest as changes in functional connectivity within networks. For this, we only looked at patients with brain tumor on the left hemisphere. Since both DMN and SN are bilateral, and the left and right ECN have component regions in the contralateral side, changes in connectivity in the right hemisphere can confidently be attributed to the tumor on the left hemisphere. We further hypothesized that these changes would correlate with, or reflect cognitive changes observed in gliomas patients. For this, we compared connectivity changes to the score of representative neuropsychometric examinations for cognition. To our knowledge, this is the first report that looked at the relationships between RSN changes and cognitive function in glioma cases. The importance of hodological understanding for these cases is also discussed.

## Methods

### Study participants

**Fig 1:**
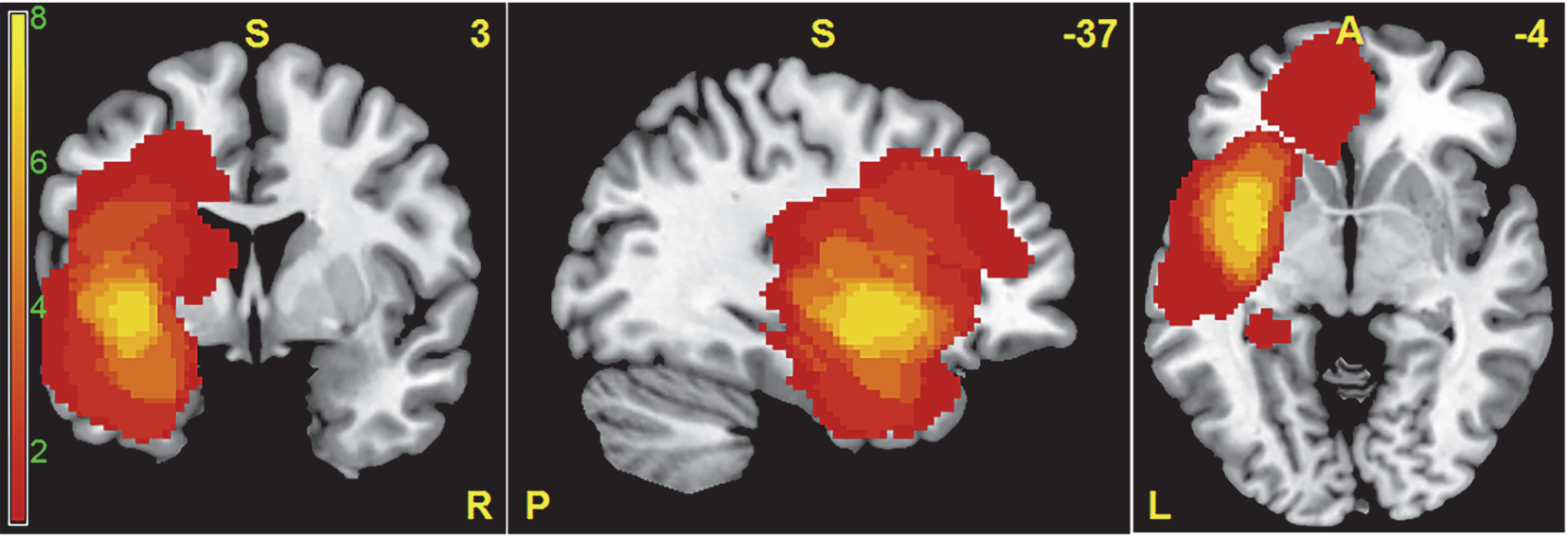
Spatial distribution of tumors in MNI standard space superimposed on a standard anatomical template. This frequency map shows the number of patients with tumor in a given voxel.

Twelve patients (mean age: 44.9 years old) with gliomas on the left hemisphere recruited from Nagoya University Hospital and 12 healthy control volunteers (mean age: 44.8 years old) were included in this study. The inclusion criteria for the patient group were as follows: 1) tumors were located in the left side, 2) tumor pathology was confirmed by surgery as gliomas, 3) the patient’s Karnofsky performance score (KPS) was greater than 80 (normal activity with effort, some signs or symptoms of disease), and 4) patient had no or minor neurological focal deficit such as speech disturbance or paresis. Tumor location is summarized in a frequency map shown in Fig. 1 and Table 1. Tumor volume, estimated by an expert neurosurgeon using each patient’s high resolution T1-weighted anatomical image and T2-weighted image, ranges from 0.6 mm^3^ to 103.5 mm^3^. The combined volume of tumor and edema was also calculated using T2-weighted images and ranged from 1.0 mm^3^ to 118.7 mm^3^. Tumor pathology was confirmed at the time of surgical removal of tumor. These characteristics are summarized in Table 1.

**Table 1:** Characteristics of patients with brain tumors.

No.	age	Tumor location	Volume (cm^3^)	volume with edema (cm^3^)	WHO grade	KPS	Speech disturbance	paresis	newly or recurrent
1	28	parietal	0.6	1.0	2	90	no	no	new
2	68	temporal	13.5	13.5	3	90	no	no	new
3	38	insula	51.5	60.2	2	100	no	no	new
4	46	insula	7.2	10.0	2	100	no	no	new
5	72	insula	25.0	25.0	2	90	no	no	new
6	34	temporal	118.7	118.7	4	90	slight	no	recurrent
7	35	insula	61.7	61.7	2	90	slight	no	recurrent
8	29	insula	103.5	113.1	3	90	slight	no	new
9	32	frontal	40.3	69.6	3	100	no	no	new
10	54	temporal	1.5	5.6	2	80	slight	no	recurrent
11	43	frontal	43.1	102.0	2	90	no	no	new
12	54	frontal	80.6	91.7	4	90	no	no	new

WHO = World Health Organization, KPS = Karnofsky performance score

In neurological examination, 4 patients had slight speech difficulty. All patients had no apparent paresis in the limbs. Lesion-related epilepsy was seen more than once in all patients. Some patients and their family reported loss of attention, decrease of memory, performance difficulty, and minor changes of characteristics. Results of cognitive testing are also listed in Table 2. Edinburgh handedness inventory was examined and confirmed that all participants were right handed. All participants have regularly taken anticonvulsants for control of epilepsy. One patient with large tumor (Number 11 in Table 1) has received corticosteroids for control of intracranial pressure.

**Table 2:** Patients’ neuropsychometric testing scores.

No.	WAIS-III							WMS-R				
	VIQ	PIQ	FIQ	VC	PO	WM	PS	VeM	ViM	GM	A/C	DR
1	118	117	120	109	121	135	100	110	116	113	128	105
2	91	105	95	92	99	88	110	83	112	92	81	74
3	83	92	86	76	106	85	75	69	83	69	103	56
4	NA	NA	NA	NA	NA	NA	NA	NA	NA	NA	NA	NA
5	112	126	120	109	119	105	130	73	102	84	99	90
6	73	79	74	71	77	83	100	69	87	71	87	<50
7	101	113	107	99	121	100	97	106	104	106	103	110
8	57	83	65	59	93	62	57	73	97	76	75	75
9	NA	NA	NA	NA	NA	NA	NA	NA	NA	NA	NA	NA
10	86	105	94	88	112	88	97	115	117	117	103	107
11	100	88	95	100	91	100	78	82	113	89	92	94
12	89	95	91	90	93	103	107	61	64	56	113	79

WAIS-III = the third version of Wechsler adult intelligence score, WMS-R = Wechsler memory scale revised, VIQ = verbal IQ, PIQ = performance IQ, FIQ = full IQ, VC = verbal comprehension, PO = perceptual organization, WM = working memory, PS = performance speed, VeM = verbal memory, ViM = visual memory, GM = generalized memory, A/C = attention/concentration, DR = delayed recall

Written informed consent was obtained from patients and healthy control volunteers prior to their participation in accordance with the study design and protocol approved by Nagoya University’s institutional review board (No. 2013-0081-2).

### Image acquisition

All MRI scans were performed using a Siemens Magnetom Verio (Siemens, Erlangen, Germany) 3.0 T scanner with a 32-channel head coil at Nagoya University’s Brain and Mind Research Center. A high resolution T1-weighted image (TR = 2.5 s, TE = 2.48 ms, 192 sagittal slices with a distance factor of 50% and 1 mm thickness, FOV = 256 mm, 256 x 256 matrix size, and an in-plane voxel resolution of 1 x 1 mm^2^) was acquired for anatomical reference from each participant. Resting state functional images were also acquired with the following imaging parameters: TR = 2.5 s, TE = 30 ms, 39 transversal slices with a 0.5 mm inter-slice interval and 3 mm thickness, FOV = 192 mm, 64 x 64 matrix dimension, flip angle = 80 degrees, and 198 volumes. Participants were instructed to close their eyes during the scan, but not to fall asleep. Other functional scans were also acquired in the same imaging session but the analysis of these datasets will be reported somewhere else. T2-weighted image (TR = 3.2s, TE = 498ms, 192 sagittal slices and 1 mm thickness, FOV = 256mm, 256 x 256 matrix size, and an in-plane voxel resolution of 1 x 1 mm^2^) was also obtained to evaluate volume of the tumor and peritumoral edema, and was used neither for preprocessing nor functional connectivity analysis.

### Image preprocessing

Functional and anatomical images were pre-processed using the FSL software package [23]. Each participant’s T1-weighted anatomical image was skull stripped using FSL’s brain extraction tool [24]. The extracted brain was then normalized to the MNI152 template using FLIRT [25]. Functional images were realigned to a representative volume from the series, co-registered to the extracted brain, and smoothed using an 8 mm full-width-at-half-maximum Gaussian filter. The smoothed functional images were then normalized to the MNI152 standard space using the same transformation matrix applied in normalizing the extracted brain image to the MNI space. The normalized images were resampled to an isotropic voxel resolution of 2 x 2 x 2 mm^3^.

Functional images were visually inspected for co-registration errors and potential image distortions after normalization due to the presence of tumor. Alignment of key anatomical landmarks such as the corpus callosum and ventricles with the reference anatomical template was used to verify proper image registration. Three patients with recurrent tumor (Numbers 6, 7, and 10 in Table 1) have titanium plate implants for cranioplasty near tumor location, darkening the image intensity in neighboring voxels. This effect was localized in areas close to the implants. For patients 6 and 8, unaffected areas surrounding the tumor were also somewhat pushed outward due to the presence of the large tumor mass. For these patients, registration of the right hemisphere, however, appeared to be mostly unaffected. For the rest of the patients, registration showed no significant deviations from the landmarks. To verify that the observed changes were not driven by one or more patients due to registration errors, regions showing significant difference in functional connectivity were further analyzed. Signals within these regions were extracted and inspected for potential outliers.

To correct for physiological noise and other nuisance signals, we regressed out the estimated motion parameters, the global signal, and mean signals from selected regions-of-interest within the cerebrospinal fluid and white matter. Analysis of the estimated motion parameters indicated no significant movement exceeding one voxel and a two-sample t-test indicated no significant difference between the mean absolute displacements of the two groups. Regressing out the global signal has been shown to artificially introduce strong negative correlation (anti-correlation) in some resting state networks [26,27]. To avoid this, negative correlations were excluded in our analysis.

### Functional connectivity analysis

The preprocessed resting state datasets from the 24 participants were temporally concatenated and group independent component analysis (ICA) using a temporal concatenation approach was performed using the MELODIC software from the FSL package. Thirty group level independent components (ICs) were extracted to be consistent with the way the RSN templates ([28], http://findlab.stanford.edu/functional_ROIs.html) we used were generated. Of the 30 extracted ICs, we selected 6 components with the greatest overlap to RSN templates of the DMN (dorsal and ventral), ECN (left and right), and SN (anterior and posterior). Differences of these networks between the patient group and healthy control group were examined using dual regression analysis [29]. In brief, the method involved using the full set of group ICs as spatial regressors in a linear model fit to obtain the temporal dynamics associated with each component for each subject. These time courses are then used as temporal regressors in a second regression analysis to estimate subject-specific spatial maps associated with each group IC. Statistical analyses of the different component maps were performed using nonparametric permutation testing with 5000 permutations for each component of interest (e.g., DMN, ECN, and SN) to identify regions showing statistically significant differences in connectivity between the patient group and the healthy control group. A threshold-free cluster enhancement technique was used to control for multiple comparisons [30]. All reported statistical maps are corrected for multiple comparisons by controlling family-wise error rate with p < 0.05.

### Seed-based connectivity analysis

We further investigated the regions showing significant changes in functional connectivity obtained in the dual regression analysis using seed-based analysis. The motivation is to identify whether such changes in RSN connectivity can be accounted by the tumor location. For this, regions showing significant difference in RSN connectivity were used as seed ROIs (region of interest). The time series from all voxels within the ROI were extracted from the same preprocessed datasets used in group ICA and the ROI’s mean series were then computed. Whole brain correlation map was generated by computing Pearson’s correlation coefficient between the mean series and the time series of all voxels within the brain. This map was then converted into a z-score map using Fisher transform and a two-sample t-test was performed to identify regions showing significant difference in connectivity between patients with brain tumor and healthy control volunteers.

### Correlation with behavioral data

**Fig 2:**
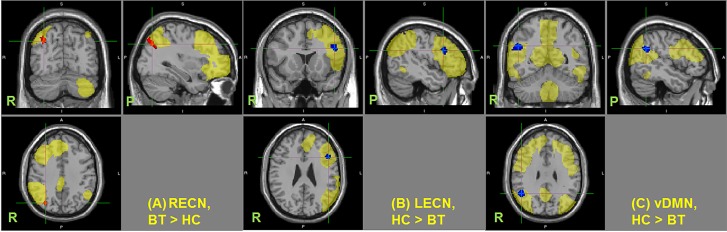
Changes in functional connectivity in resting state networks. Regions showing significant changes in functional connectivity in the (A) right executive control network (RECN), (B) left executive control network (LECN), and (C) ventral default mode network (vDMN) (p < 0.05 TFCE-cluster corrected for multiple comparison). HC–healthy control, BT–patients with brain tumor. Resting state networks are shown in yellow. Center of the cursor locates maximum significance.

We assessed the significance of the changes in functional connectivity to the patients’ cognitive function as measured by the patient’s neuropsychometric evaluation score. For neuropsychometric evaluation, the third edition of the Wechsler Adult Intelligence scale (WAIS) and revised edition of Wechsler Memory Scale (WMS-R) were performed in brain tumor cases. WAIS-III is designed to measure intelligence in adults and older adolescents, which provides the scores of verbal intelligence quotient (VIQ), performance IQ (PIQ) and full scale IQ (FIQ), with four secondary indices including verbal comprehension, working memory, perceptual organization, and processing speed [31]. WMS-R is designed to measure different memory functions in a person, including verbal memory quotient, visual memory quotient, general memory quotient, attention/concentration quotient, and delayed recall quotient [32]. The index scores of WAIS-III and WMS-R have a mean of 100, and standard deviation of 15. Two of the patients didn’t have the behavioral data so we excluded them in this analysis. ROIs were generated from the regions showing significant difference in functional connectivity between the patient group and the healthy control group (see Fig. 2A-C). Connectivity measures were then extracted from these ROIs using the subject-specific RSN maps computed in the back reconstruction stage. The extracted values were averaged from all voxels within the ROI and average value correlated with the behavioral measures using Pearson’s correlation. For reference, the correlation with all other measures, such as age, tumor volume, combined volume of tumor and peritumoral edema, WHO grading, and KPS, were evaluated as well.

## Results

### Dual regression analysis

Of the three RSNs investigated, only three showed significant difference in functional connectivity between the healthy control group and the patient group. Fig. 2 shows the regions with altered connectivity in the patient group for the right ECN (Fig. 2A), left ECN (Fig. 2B), and DMN (Fig. 2C). Ventral DMN showed significant decrease in functional connectivity in the right angular gyrus/inferior parietal lobe in patients with brain tumor compared to healthy controls. The right ECN also showed significant increase in connectivity around the right intraparietal sulcus in the patient group compared to the control group, whereas a significant decrease in connectivity in the left ECN was observed in the left DLPFC (Table 3).

**Table 3:** Peak locations of regions showing significant difference in functional connectivity in the 3 resting state networks investigated.

Resting State Network	Areas	Peak Location(MNI Coordinates)			p-value (corrected)	Cluster Size (voxels)
		X	Y	Z		
Ventral Default Mode Network (healthy > patient)	Right angular gyrus / inferior parietal lobule	50	-54	30	0.004	163
Right Executive Control Network (healthy < patient)	Right intraparietal sulcus	34	-72	38	0.006	138
Left Executive Control Network (healthy > patient)	Left middle frontal gyrus / dorsolateral prefrontal cortex	-46	16	26	0.015	126

Reported coordinates are in MNI standard space.

It should be noted that two of the patients had tumors intersecting this region, which could potentially affect the estimation of the connectivity measure and the corresponding statistical test. To verify this, we did an ROI analysis to check if the obtained values are significantly different from the rest of the group (outliers). The result showed that the mean connectivity within the region for the two patients didn’t significantly differ from those of the rest of the patients. Finally, analysis of the connectivity of both the anterior and posterior salience networks didn’t show any significant change between the two populations.

Following previous reports [20,21], we also compared connectivity changes between low grade (7 participants, WHO grade 2) and high grade (5 participants, WHO grade 3 and 4) glioma patients. Results showed no significant difference in functional connectivity in all three networks investigated even when using an uncorrected p-value < 0.001.

### Seed-based analysis

**Fig 3:**
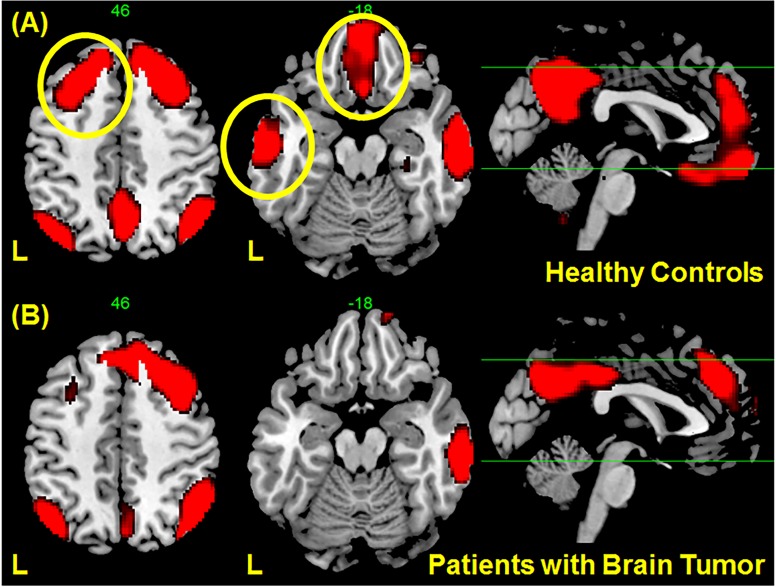
Regions correlated with the right angular gyrus (Fig. 2C) obtained using seed-based connectivity analysis in (A) healthy controls and (B) patients with gliomas. Correlations in several regions in the left hemisphere, indicated by yellow circle in (A), are no longer significant in patients with gliomas although only the region in the middle temporal gyrus showed statistical significance. The maps showed voxels with t values < 3.5.

To investigate further whether the change in connectivity of the vDMN in the right angular gyrus was due to the location of the tumor, seed-based analysis was performed. Results of the whole-brain connectivity analysis with the right angular gyrus (Fig. 2C) as the seed region indicated significant changes in connectivity in the left hemisphere (see Fig. 3). A number of regions in the left hemisphere showed strong correlation with the seed region in the healthy control group as shown in Fig. 3A (indicated by the yellow circles). On the other hand, the correlation of these regions with the seed region significantly decreases in the patient group as shown in Fig. 3B, but not the connectivity on the ipsilateral side. In particular, a significant decrease in connectivity in the left middle temporal gyrus was observed. This loss of functional connectivity from these regions to the right angular gyrus in patients with brain tumor as compared to healthy controls could be due to the presence of the tumor in this hemisphere.

### Correlation analysis

**Fig 4:**
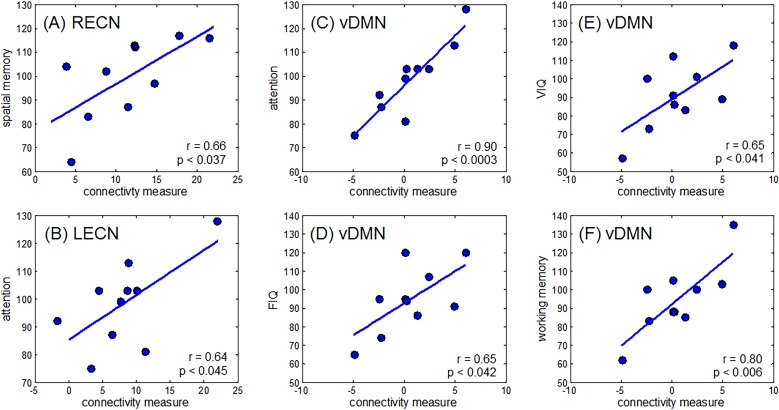
Relationship between changes in connectivity and several cognitive measures. Connectivity measures were extracted from regions shown in Fig. 2. Strong correlations were observed between changes in (A) RECN and spatial memory, (B) LECN and attention, (C) vDMN and attention, (D) vDMN and FIQ, (E) vDMN and VIQ, and (F) vDMN and working memory.

To examine the relevance of the changes in RSN connectivity in the patient group, an ROI correlation analysis between RSN connectivity and neuropsychometric evaluation scores was performed. Results are shown in Table 4 and Fig. 4.

**Table 4:** Correlation coefficients (r-value) with behavioral data and patient characteristics.

	WAIS-III							WMS-R				Other Measures				
	VIQ	PIQ	FIQ	VC	PO	WM	PS	VeM	ViM	GM	A/C	Age	WHO	Vol	Vol2	KPS
RECN	0.06	0.06	0.07	0.04	0.09	0.18	-0.15	0.52	0.66	0.57	0.09	-0.21	-0.22	-0.43	-0.39	-0.01
LECN	0.41	0.48	0.46	0.31	0.45	0.59	0.36	0.33	0.11	0.30	0.64	-0.03	-0.08	-0.50	-0.68	0.28
vDMN	0.65	0.56	0.65	0.60	0.54	0.80	0.47	0.30	-0.14	0.19	0.90	0.04	-0.11	-0.43	-0.51	0.15

Underlined correlation coefficients have p-values < 0.05. Abbreviations defined in Table 2. Vol = tumor volume, Vol2 = combine volume of tumor and edema

Changes in connectivity measures in the right ECN correlated with spatial memory (r = 0.66, p < 0.037), while that on the left ECN correlated with attention (r = 0.64, p < 0.045). Moreover, connectivity changes in the ventral DMN also correlated with attention (r = 0.90, p < 0.0003), working memory (r = 0.80, p < 0.006), FIQ (r = 0.65, p < 0.042), and VIQ (r = 0.65, p < 0.041) measures in the patient group.

Aside from cognitive scores, only the combined volume of tumor and edema showed statistically significant negative-correlation to connectivity changes in the left ECN (r = -0.68, p < 0.03). All other measures, such as age, tumor volume, WHO grading, and KPS do not reach statistically significant correlation to connectivity changes in the right ECN, left ECN, or ventral DMN (Table 4).

## Discussion

Resting state functional MRI has played a key role in identifying the different intrinsic functional networks within the brain. In this study, we examined three well-known RSNs—which includes the DMN, ECN, and SN—in glioma patients and found significant changes in functional connectivity in the patient group compared to healthy controls in two of the three networks investigated. Moreover, the changes in connectivity correlated with the patients’ cognitive function as measured by the scores of their neuropsychometric evaluation. This could potentially explain some of the behavioral changes often seen in glioma cases, such as slowness of reaction, somnolence, loss of attention, and working memory problems.

Statistically significant changes were observed in the right angular gyrus/inferior parietal lobule (Fig. 2A) of the DMN. This is consistent with our hypothesis that unilateral tumors influenced the connectivity of the intact contralateral hemisphere. Additional seed-based connectivity analysis using this region as the seed point showed significant decrease in connectivity to the left frontal and temporal areas in the patient group (Fig. 3). Since some tumors were located in these areas, the decrease in connectivity of the DMN was likely a consequence of the damage of the network in the left hemisphere. This finding suggests that network disruption is not confined in the tumor’s location. Distant cortical or subcortical regions connected around or within the tumor are also affected. Surprisingly, we did not observe significant changes in connectivity in the left hemisphere. The reason could be that the effect on the left side varies from patient to patient, depending on the tumor location, such that the group effect is less evident and does not reach statistical significance. On the other hand, the effect on the right hemisphere is consistent among all patients leading to the observed statistical significance.

Our finding is also in general agreement with results from earlier studies [20,21] that found similar alterations in the DMN in patients with gliomas, although some differences in details such as locations or direction of change exist. However, these could be explained by the differences in methodology as well as the type of datasets used.

Of novel importance in this study in comparison to previous reports is the correlation we found between DMN connectivity of the affected area and the cognitive function measured by cognitive evaluation scores. In general, it was indicated that there was a strong positive association between the global efficacy of functional brain networks and intelligence performance in healthy participants [33] and in brain tumor cases [22]. Although the patients showed no apparent neurological deficits such as aphasia and paresis, cognitive test scores including WAIS-III or WMS-R showed some degree of cognitive decline in some cases. We found that the integrity of the DMN positively correlated with the score of the VIQ and FIQ in the WAIS-III, the working memory, and attention (Fig. 4C-D). Although decreased integrity was seen in the right angular gyrus in the DMN, which showed decreased connectivity to the left frontal and temporal areas where the language center usually is located (Fig. 3), correlation to decrease of VIQ could therefore reflect neural network alteration due to lesional damage of the left cortices.

Aside from the observed connectivity changes in DMN, we also found a decrease in integrity of the left ECN, as well as an increase in the right ECN. The changes observed in the left ECN were localized in the left DLPFC (Fig. 2B). This is understandable considering the fact that glioma patients often show loss of attention, reduction of performance ability, and deterioration of working memory. Supporting this hypothesis is the observed significant correlation between the reduced attention score in WMS and decreased connectivity in the left ECN (Fig. 4B). This decrease in connectivity in the left ECN was also correlated to the combined volume of the tumor and edema (Table 4). The edema formed by the tumor spreads in the white matter (i.e., subcortical networks), which could disrupt neighboring local network, thus, decreasing the connectivity of the left ECN. On the other hand, the score of the spatial memory correlated with connectivity increases in the right ECN (Fig. 4A), which could be viewed as a compensation mechanism where the spatial memory function in the right ECN compensates for the reduction in verbal memory and cognition in the left ECN.

Contrary to expectation, no significant change was observed in the salience network. Considering global changes of the neural networks in glioma patients, we expected that the SN should also decrease in the same way as the other two networks. The right anterior insula, an important node of the SN, plays a crucial and causal role in switching the two other major networks (ECN and DMN). Since all subjects had their tumors in the left hemisphere, their right anterior insula remained intact, thereby possibly preserving the integrity of the salience network.

Finally, we could not find statistically significant correlation between tumor locations and cognitive functions probably due to the small number of patients in this study. However, we’ve seen certain trends in the available behavioral dataset. Large tumors located in both frontal and temporal lobes tended to decrease function related to language, such as VIQ, verbal comprehension, and verbal memory. Tumors in the temporal lobes extending to the insula tended to affect verbal memory. In general, working memory and attention can be affected by tumors located in the frontal lobe (the dorsolateral prefrontal cortex, medial prefrontal cortex, the anterior cingulate cortex) and/or the parietal lobe (the inferior parietal lobule). However, patients with frontal or parietal lobe tumors included in this study didn’t show decline in working memory or attention. To fully understand changes of cognition, we presumed that not only topological but also network-based (hodological) approaches are necessary.

### Limitations

One limitation of this study is the small number of participants, which prevented us from detecting smaller effect sizes and could also explain the null result we obtained for the salience network. Consequently, we were also unable to detect significant changes in functional connectivity between pathological grading types as has been previously done. This comparison could have provided additional insights into this issue as previous papers had shown differing views.

Another issue we were unable to examine was the effect of tumor location. It would be interesting to know how tumors on the right side could affect the observed connectivity changes in the right hemisphere or could lead to connectivity changes in the left hemisphere. Comparisons between the tumor location (right or left, frontal or temporal, etc.) could provide additional insights into our hodological understanding of brain functions in glioma patients.

There may also be a technical limitation in the evaluation of the functional connectivity in the same hemisphere as the tumor’s location since the tumor mass could affect the statistical calculation in the group analysis. This is probably the reason why we didn’t observe obvious connectivity changes in the DMN in the left hemisphere. However, the evaluation of contralateral side should not be affected by this.

## Conclusion

In conclusion, we found changes in RSNs in patients with gliomas who showed no apparent aphasia or paresis in limbs, but with observable changes in cognitive function. Connectivity changes were observed mostly on the contralateral hemisphere supporting our hypothesis. Moreover, these alterations were correlated with the observed changes in patients’ cognitive function as measured by neuropsychometric testing. Evaluation of resting state networks is therefore potentially helpful in advancing our hodological understanding of brain function in glioma cases.

## References

[1] Mahaley MS, Mettlin C, Natarajan N, Laws ER, Peace BB (1989) National survey of patterns of care for brain-tumor patients. J Neurosurg 71: 826–836. 10.3171/jns.1989.71.6.0826 2585073

[2] Sporns O (2011) The human connectome: A complex network. Ann N Y Acad Sci 1224: 109–125. 10.1111/j.1749-6632.2010.05888.x 21251014

[3] Greicius MD, Krasnow B, Reiss AL, Menon V (2003) Functional connectivity in the resting brain: a network analysis of the default mode hypothesis. Proc Natl Acad Sci U S A 100: 253–258. 10.1073/pnas.0135058100 12506194 PMC140943

[4] Damoiseaux JS, Rombouts SARB, Barkhof F, Scheltens P, Stam CJ, et al (2006) Consistent resting-state networks across healthy subjects. Proc Natl Acad Sci U S A 103: 13848–13853. 10.1073/pnas.0601417103 16945915 PMC1564249

[5] Chen S, Ross TJ, Zhan W, Myers CS, Chuang KS, et al (2008) Group independent component analysis reveals consistent resting-state networks across multiple sessions. Brain Res 1239: 141–151. 10.1016/j.brainres.2008.08.028 18789314 PMC2784277

[6] Bettus G, Bartolomei F, Confort-Gouny S, Guedj E, Chauvel P, et al (2010) Role of resting state functional connectivity MRI in presurgical investigation of mesial temporal lobe epilepsy. J Neurol Neurosurg Psychiatry 81: 1147–1154. 10.1136/jnnp.2009.191460 20547611

[7] Lee MH, Smyser CD, Shimony JS (2013) Resting-state fMRI: a review of methods and clinical applications. Am J Neuroradiol 34: 1866–1872. Available: http://www.ncbi.nlm.nih.gov/pubmed/22936095. 10.3174/ajnr.A3263 22936095 PMC4035703

[8] Kokkonen SM, Nikkinen J, Remes J, Kantola J, Starck T, et al (2009) Preoperative localization of the sensorimotor area using independent component analysis of resting-state fMRI. Magn Reson Imaging 27: 733–740. 10.1016/j.mri.2008.11.002 19110394

[9] Sridharan D, Levitin DJ, Menon V (2008) A critical role for the right fronto-insular cortex in switching between central-executive and default-mode networks. Proc Natl Acad Sci U S A 105: 12569–12574. 10.1073/pnas.0800005105 18723676 PMC2527952

[10] Uddin LQ, Menon V (2009) The anterior insula in autism: Under-connected and under-examined. Neurosci Biobehav Rev 33: 1198–1203. 10.1016/j.neubiorev.2009.06.002 19538989 PMC2743776

[11] McKiernan KA BR, Kaufman JN, Binder JR (2006) Interrupting the “stream of consciousness”: An fMRI investigation. Neuroimage 29: 1185–1191. 10.1016/j.neuroimage.2005.09.030 16269249 PMC1634934

[12] Goldberg II, Harel M, Malach R (2006) When the Brain Loses Its Self: Prefrontal Inactivation during Sensorimotor Processing. Neuron 50: 329–339. 10.1016/j.neuron.2006.03.015 16630842

[13] Spreng RN, Mar RA, Kim ASN (2009) The common neural basis of autobiographical memory, prospection, navigation, theory of mind, and the default mode: a quantitative meta-analysis. J Cogn Neurosci 21: 489–510. 10.1162/jocn.2008.21029 18510452

[14] Supekar K, Menon V, Rubin D, Musen M, Greicius MD (2008) Network analysis of intrinsic functional brain connectivity in Alzheimer’s disease. PLoS Comput Biol 4: e1000100 Available: http://www.ncbi.nlm.nih.gov/pubmed/18584043. 10.1371/journal.pcbi.1000100 18584043 PMC2435273

[15] Zhu DC, Majumdar S, Korolev IO, Berger KL, Bozoki AC (2013) Alzheimer’s disease and amnestic mild cognitive impairment weaken connections within the default-mode network: A multi-modal imaging study. J Alzheimer’s Dis 34: 969–984. 10.3233/JAD-121879 23313926

[16] Waltz JA, Kasanova Z, Ross TJ, Salmeron BJ, McMahon RP, et al (2013) The Roles of Reward, Default, and Executive Control Networks in Set-Shifting Impairments in Schizophrenia. PLoS One 8 10.1371/journal.pone.0057257 23468948 PMC3584128

[17] Filippi M, Agosta F, Scola E, Canu E, Magnani G, et al (2013) Functional network connectivity in the behavioral variant of frontotemporal dementia. Cortex 49: 2389–2401. 10.1016/j.cortex.2012.09.017 23164495

[18] Koechlin E, Hyafil A (2007) Anterior prefrontal function and the limits of human decision-making. Science 318: 594–598. 10.1126/science.1142995 17962551

[19] Menon V, Uddin LQ (2010) Saliency, switching, attention and control: a network model of insula function. Brain Struct Funct: 1–13. 10.1007/s00429-010-0262-0. PMC2899886 20512370

[20] Harris RJ, Bookheimer SY, Cloughesy TF, Kim HJ, Pope WB, et al (2014) Altered functional connectivity of the default mode network in diffuse gliomas measured with pseudo-resting state fMRI. J Neurooncol 116: 373–379. 10.1007/s11060-013-1304-2 24234804 PMC6763342

[21] Esposito R, Mattei PA, Briganti C, Romani GL, Tartaro A, et al (2012) Modifications of default-mode network connectivity in patients with cerebral glioma. PLoS One 7 10.1371/journal.pone.0040231 22808124 PMC3392269

[22] Xu H, Ding S, Hu X, Yang K, Xiao C, et al (2013) Reduced efficiency of functional brain network underlying intellectual decline in patients with low-grade glioma. Neurosci Lett 543: 27–31. 10.1016/j.neulet.2013.02.062 23562503

[23] Jenkinson M, Beckmann CF, Behrens TEJ, Woolrich MW, Smith SM (2012) FSL. Neuroimage 62: 782–790. 10.1016/j.neuroimage.2011.09.015 21979382

[24] Smith SM (2002) Fast robust automated brain extraction. Hum Brain Mapp 17: 143–155. 10.1002/hbm.10062 12391568 PMC6871816

[25] Jenkinson M, Bannister P, Brady M, Smith S (2002) Improved optimization for the robust and accurate linear registration and motion correction of brain images. Neuroimage 17: 825–841. 10.1016/S1053-8119(02)91132-8 12377157

[26] Murphy K, Birn RM, Handwerker DA, Jones TB, Bandettini PA (2009) The impact of global signal regression on resting state correlations: Are anti-correlated networks introduced? Neuroimage 44: 893–905. 10.1016/j.neuroimage.2008.09.036 18976716 PMC2750906

[27] Van Dijk KRA, Hedden T, Venkataraman A, Evans KC, Lazar SW, et al (2010) Intrinsic functional connectivity as a tool for human connectomics: theory, properties, and optimization. J Neurophysiol 103: 297–321. 10.1152/jn.00783.2009 19889849 PMC2807224

[28] Shirer WR, Ryali S, Rykhlevskaia E, Menon V, Greicius MD (2012) Decoding subject-driven cognitive states with whole-brain connectivity patterns. Cereb Cortex 22: 158–165. Available: http://www.ncbi.nlm.nih.gov/pubmed/21616982. 10.1093/cercor/bhr099 21616982 PMC3236795

[29] Filippini N, MacIntosh BJ, Hough MG, Goodwin GM, Frisoni GB, et al (2009) Distinct patterns of brain activity in young carriers of the APOE-ε4 allele. Proc Natl Acad Sci 106: 7209–7214. Available: http://www.pnas.org/content/106/17/7209.abstract. 10.1073/pnas.0811879106 19357304 PMC2678478

[30] Nichols TE, Holmes AP (2002) Nonparametric permutation tests for functional neuroimaging: A primer with examples. Hum Brain Mapp 15: 1–25. Available: http://www.ncbi.nlm.nih.gov/pubmed/11747097. 11747097 10.1002/hbm.1058 PMC6871862

[31] Ryan JJ, Schnakenberg-Ott SD (2003) Scoring reliability on the Wechsler Adult Intelligence Scale-Third Edition (WAIS-III). Assessment 10: 151–159. 10.1177/1073191103010002006 12801187

[32] Elwood RW (1991) The Wechsler Memory Scale-Revised: psychometric characteristics and clinical application. Neuropsychol Rev 2: 179–201. 10.1007/BF01109053 1844708

[33] Van den Heuvel MP, Stam CJ, Kahn RS, Hulshoff Pol HE (2009) Efficiency of functional brain networks and intellectual performance. J Neurosci 29: 7619–7624. 10.1523/JNEUROSCI.1443-09.2009 19515930 PMC6665421

